# Tactile Sensing Using Magnetic Foam

**DOI:** 10.3390/polym14040834

**Published:** 2022-02-21

**Authors:** Gildas Diguet, Joerg Froemel, Masanori Muroyama, Koichi Ohtaka

**Affiliations:** 1 Division for the Establishment of Frontier Sciences, Organization for Advanced Studies, Tohoku University, 2-1-1 Katahira, Aoba-ku, Sendai 980-8577, Japan; joerg.froemel.e5@tohoku.ac.jp (J.F.); muroyama@tohoku.ac.jp (M.M.); 2 Advanced Institute for Materials Research, Tohoku University, 2-1-1 Katahira, Aoba-ku, Sendai 980-8577, Japan; 3 Department of Robotics, Graduate School of Engineering, Tohoku University, 6-6-01, Aza Aoba, Aramaki Aoba-ku, Sendai 980-8579, Japan; 4 Department of Electrical and Electronic Engineering, Faculty of Engineering, Tohoku Institute of Technology, Yagiyama Kasumi-cho 35-1, Taihaku-ku, Sendai 982-8577, Japan; 5 Micro System Integration Center, Tohoku University, 519-1176 Aza Aoba, Aramaki Aoba-ku, Sendai 980-0845, Japan; koichi.ohtaka.e8@tohoku.ac.jp

**Keywords:** magnetic composite, deformation sensors, artificial skin

## Abstract

For biomedical applications, smart materials that are used as sensors or actuators have to match some criteria, especially bio-compatibility and softness. Smart polymers are candidates that fulfill these two criteria. A sensitivity to compression is created by adding magnetic particles to a compressible foam polymer. A foam-based composite is fabricated for its small Poisson’s ratio, which enables significant compression, up to 50%. This large compression induces a change in its magnetic properties, which can be detected using coils. By setting the sensing coils as a compact array of 3 × 3, the sensor successfully detected and localized an applied deformation.

## 1. Introduction

For some applications where sensing or actuation is needed, it is important to avoid rigid materials, such as soft actuators for biomedical applications [1]. Artificial muscle highlights the importance of compliance compatibility: the actuator should not damage the soft biological body, and this condition was satisfied by the choice of this silicone matrix. The actuation of the silicone rubber was tuned by the thermal expansion of liquid-vapor fillers. These smart materials based on polymers have several major advantages over piezoelectric materials or magnetostrictive ceramic materials: they are easy to process by a chemical reaction between a few components, they can be molded or 3D-printed, they are cheap, they are deformable (stretchable, compressive, flexible, etc.) [2], they have a low Young’s modulus (from 0.001 to 1 Gpa), they are light-weighted materials, and they are bio-compatible.

Smart polymers can be used in the reverse function: as a sensing material. A typical soft composite sensor technology relies on its electrical conductivity (or resistivity). Electrical conductivity is a function of the filling factor *ϕ*_v_ of the composite [3], defined as the ratio of the filler volume divided by the sample volume, and beyond the percolation threshold *ϕ*_c_ the composite conductivity increases drastically. By compressing the composite, the electric conductivity then increases [4]. The largest electrical conductivity increase is found in a small compression range window: close to the percolation threshold.

By using magneto-rheological elastomers (MREs), which can provide a good magneto-elastic coupling [5], an inductive sensing technology can be obtained. A system made of a bilayer material on top of an array of coils [6], or a magnetic field sensor [7], was used to detect deformation; the bilayer was composed of an MRE as a top layer and pure silicon rubber as a bottom layer [6,7,8]. A signal was detected as the pressure applied to the top MRE pushed this magnetic layer closer to the field sensitive element. The dimensions in [6,7] were large; in this bilayer system, the MRE thickness was 3 mm [6] or 2 mm [7], plus the pure Silicone Rubber thickness was 10 mm. The coil array system used in [6] was also large; each coil had a diameter of 10 mm separated by a gap of 15 mm. In the case of [7], the field sensors were distanced by 30 mm.

In this article, a more compact system is designed. A single MRE layer is used as the sensing part of the sensor. Instead of using a continuous matrix, like the usual silicone rubber of MRE, a porous foam is used as the host matrix. Porosity in materials is known to reduce the effective Young’s modulus [9,10]; the composite is then softer. Polymeric foams, such as polyurethane, are highly compressible thanks to their Poisson’s ratio, which can be as low as 0.1 [11]. The reason for the choice of this matrix is to achieve greater compression than that achievable with silicone rubber. A composite based on a porous matrix is then an interesting direction for the tenability of the smart properties of composite over a large range of deformation, especially in the case of sensing capability. Some applications of magnetic foam already exist, such as microwave absorption [12] or the energy-harvesting system patch on the human body, composed of a porous silicone filled by hard magnetic particles, NbFeB, powering a thermometer for continuous health monitoring [13,14]. The magnetic remnant state of the composite is changed by a mechanical stimulus. This system relied on hard magnetic particles (NdFeB particles) and had to be initially magnetized in a highly magnetic field (2.6 T). On the other hand, the magnetic permeabilities of magnetic composite using soft magnetic fillers, such as Fe particles, were also seen to vary because of compression [15], and a simple model was provided for the magnetic foam properties’ variation with compression. The magnetic properties are a function of the filling factor, and as this magnetic foam volume is reduced by an applied force, its content of magnetic material and permeability are increased. Such an increase in magnetic permeability can be detected by a sensing coil.

The deformation/force sensor device studied in this article uses a unique and simple smart layer: bilayer composites using MRE materials used to sense applied deformation [6,7]. Combined with a compact coil array of 3 × 3 coils on a 20 × 20 mm^2^ surface, the device can detect the point of pressure application; hence, it can mimic the tactile sense of the skin. Deformation up to 50% was recorded and successfully localized. The equivalent force range detected was up to 4.5 N with a resolution of 0.29 N.

## 2. Materials and Methods

### 2.1. Magnetic Foam Composite Preparation

**Figure 1:**
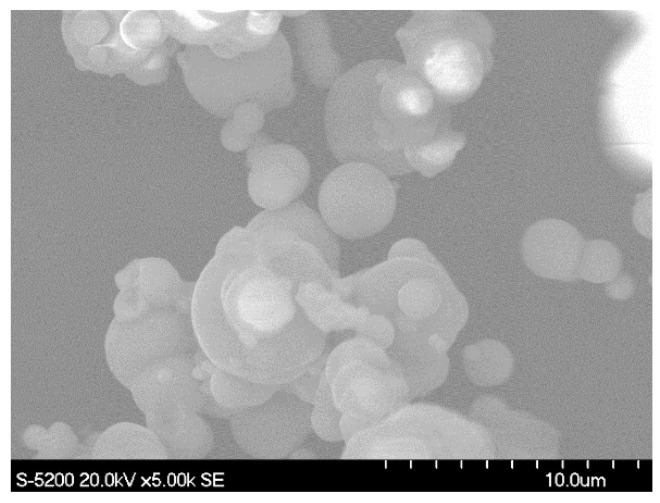
SEM picture of the magnetic particles.

**Figure 2:**
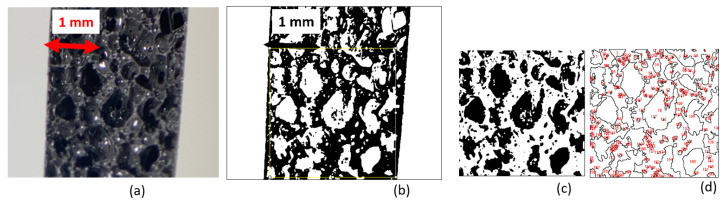
Sample cross-section (**a**), threshold processed picture (**b**), picture analysis selection (yellow square), color inversion of the selected area (**c**), and the result of the picture analysis with the number tag corresponding to the black area on the picture (**d**).

The experimental materials consisted of a polymer foam, filled with magnetic particles. These magnetic particles were spherical carbonyl-iron particles (CIP-CS, BASF, Japan) with a diameter D50 = 6.0–7.0 µm. Figure 1 shows an image of these particles by using a field emission scanning electron microscope (FE-SEM; S-5200 from Hitachi). The host matrix was a commercial polyurethane foam (CM-218, PROST, Japan) as a bi-component product. Particles were mixed into the foam with weight fraction; *ϕ*_m_ = m_Fe_/m_tot_ = 70%; m_Fe_ and m_tot_ are the particles’ mass and the total mass, respectively. Particles were first mixed with the solution and hand-stirred for 5 min; then the curing agent was added. The mixture was molded as a membrane with a controlled thickness, a 3.1 mm thick sample. The curing time was about 20 min at room temperature. Once cured, the membrane was cut into 20 × 20 mm^2^. Figure 2 presents the internal pore structure of the obtained sample. The remaining parts were used for characterizing the mechanical and magnetic properties.

The composite expanded and large pores were formed with size of up to 1 mm. By image analysis, performed using ImageJ software analysis [16], the picture was converted into a binary image using the threshold function, Figure 2b, by measuring the black and white area surfaces, Figure 2c,d; the ratio between the black and white colors provided the information on the porosity [17]. A ratio close to 45% was obtained to measure the porosity.

### 2.2. Sensing Device

**Figure 3:**
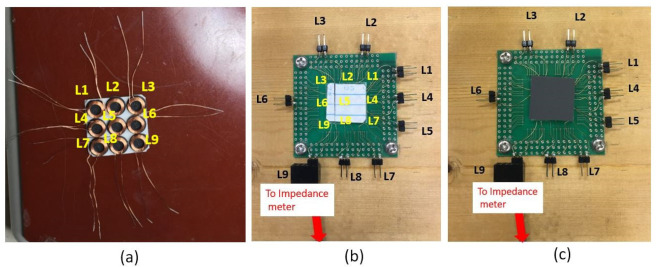
Coils array device fabrication: (**a**) 3 × 3 coils, (**b**) array glued on the PCB, and (**c**) with a 20 × 20 mm^2^ sample. Here the inductance L9 is connected to the impedance meter for measurement.

The composite is the element that reacts to the deformation. For the detection, an array of nine coils was used. Each coil acts as a touch-sensitive site, or taxtel [18]. These coils, purchased from RS components, were 2 mm in height with a diameter of *d* = 6 mm and with an initial inductance of 7.2 µH. The nine coils were placed into a 3 × 3 array with dimensions of 20 × 20 mm^2^, as seen in Figure 3a. Taxtel size is 20/3~6.7 mm, which is smaller than 7.5 mm in [6] or 30 mm [7]. The array was glued onto a PCB to fix this device to connectors. Each coil was then labeled L1 to L9 for tracking the deformation by the measurement of the change of inductance (Figure 3b). Finally, the foam was placed above the array (Figure 3c).

The measurement of the inductance of each coil was performed at different levels of deformation. Inductance was measured using an impedance meter (PSM1735 from Newtons4th, Newtons4th) in LCR mode at the frequency of 20 kHz. Uncertainty on the inductance measurement was 1 nH.

The deformation was applied using a three-axis stage from COMS (PM80B-50Z and PM80B-100XY-C, COMS, Japan), and the pressure tip was 3D-printed with ABS to avoid any eddy current interference from any metallic part. The deformation area was chosen by positioning the tip over the XY plane. The deformation was obtained by pushing the tip down (Z axis) into the composite by steps of ΔZ = −100 µm.

### 2.3. Mechanical Testing

The mechanical properties of the sample were measured with a tensile/compression tester (Zwitckline Z0.5TN, ZWICK). The sample was placed between two plates to apply the compression. Force was applied with a rate of 4 mN/s with steps of 0.2 N up to 2 N. Displacement and force were recorded during this compression test. Engineering stress was then obtained from the measured force *F* divided by the initial specimen cross-sectional area (*A*_0_ = 3.55 mm^2^) as [8,19]:

(1) $$\sigma =\frac{F}{A_{0}}$$

The compressive engineering strain was obtained from the measured displacement Δ*t* divided by the initial specimen thickness (*t*_0_ = 3.1 mm) as [8,19]:

(2) $$\varepsilon =\frac{\Delta t}{t_{0}}$$

The change of thickness Δ*t* is taken as negative for compressive tests, so the compression *ε* defined by Equation (2) is also negative in this article.

### 2.4. Magnetic Characterization

The sample was tested with a vibrating sample magnetometer (VSM BHV-50H, Riken Denshi, Japan) for measuring the magnetization curve of ±10 kOe (~800 kA/m). Before the sample experimental measurement, a Ni sample was first measured to adjust the equipment settings to match with the theoretical magnetic moment of the Ni calibration sample. After this calibration procedure, the magnetization loop of the magnetic composite was performed. By adjusting the clamping system, a deformation could be applied to the sample for the magnetic measurement.

## 3. Results

### 3.1. FTIR

**Figure 4:**
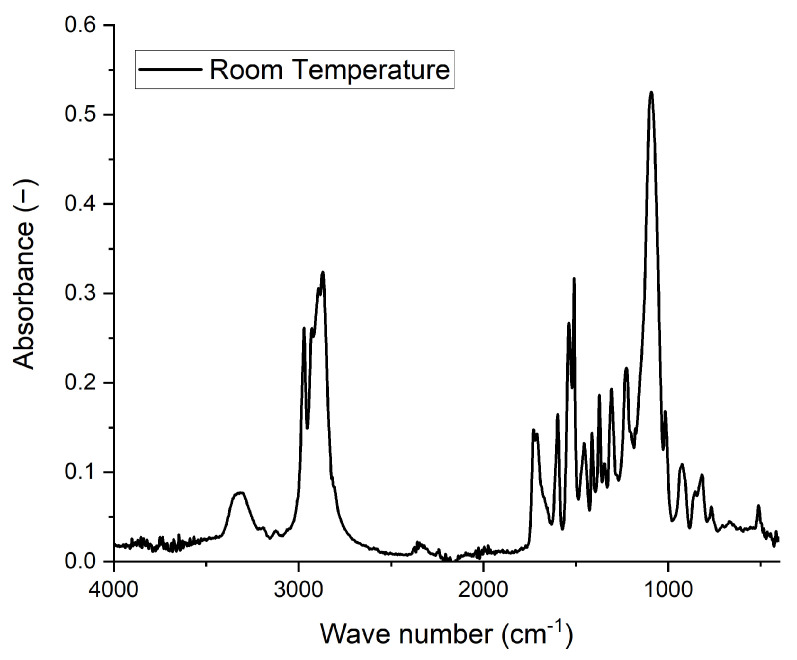
Room temperature absorbance spectrum of the polyurethane.

The absorbance spectrum measured on the pure foam is presented in Figure 4. Several characteristics peaks observed in Figure 4 assert the foam’s chemical nature as a polyurethane. The corresponding peaks, and their corresponding physical interpretation, are gathered in Table 1.

### 3.2. Mechanical Test

**Figure 5:**
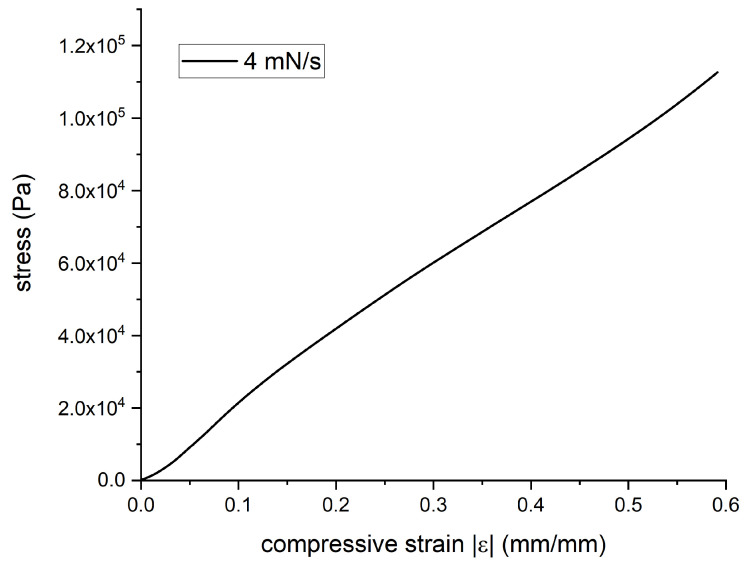
Stress versus strain curve in compression.

The composite force versus displacement was measured, and the obtained stress versus strain plot is presented in Figure 5.

From this strain-stress curve, the linear approximation provided the value of Young’s modulus of *E* = 200 kPa, which corresponds to soft materials such as elastomers, especially porous polymers.

### 3.3. Magnetization Loop

**Figure 6:**
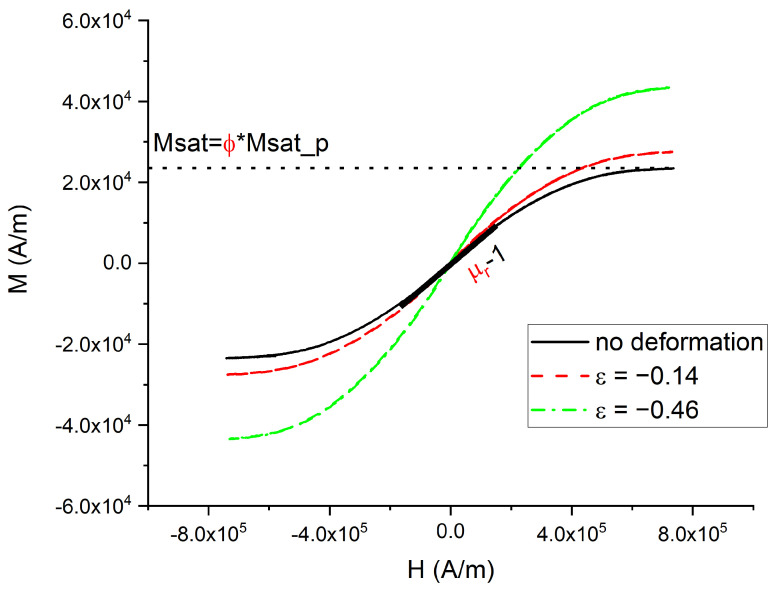
Magnetization versus applied field for different compression. Bold line and dotted lines highlight the magnetic permeability and saturation of the non-deformed composite, respectively.

The magnetization versus magnetic field is presented in Figure 6.

For the measurement with no deformation applied, the curve did not present any magnetic hysteresis because of the very soft magnetic properties of the CIP-CS particles. This curve also provided the information about the magnetic quantity inside the material; the magnetic saturation *Msat* is directly proportional to the magnetic particles’ magnetic saturation, *Msat_p* = 1.72 MA/m [23]. The proportional coefficient corresponds to the filling factor *ϕ*_v_. The filling factor is defined as the volume represented by all the CIPs (n*V_p_, with *n* the particle number with individual volume *V*_p_) inside the sample volume (*V*_tot_) as:

(3) $$\phi =\frac{nVp}{V\mathrm{tot}}$$

The corresponding value of *Msat* = 23,500 A/m pointed to a filling factor of *ϕ*_v_ = 1.35%. Sample magnetization was also recorded at a different level of compression ε defined according to Equation (2).

**Figure 7:**
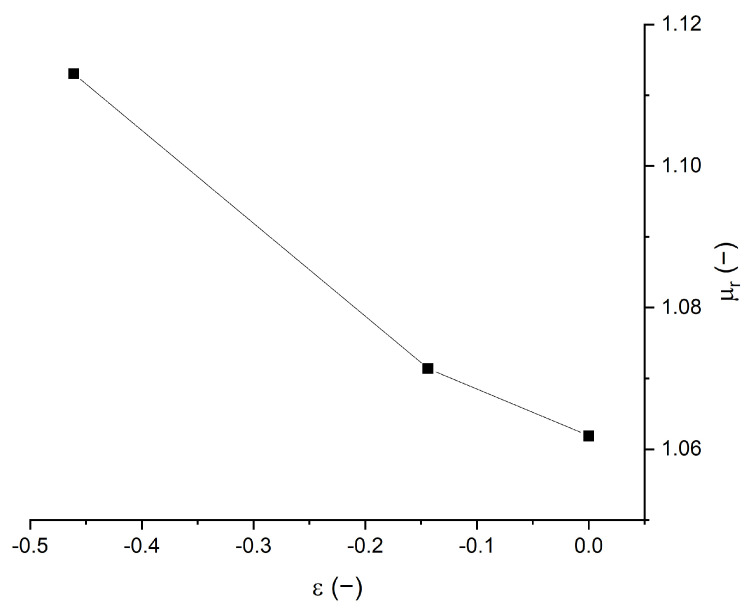
Magnetic relative permeability versus compression strain.

The magnetization loop is affected by the compressive strain. The compression yields to an increase in the magnetic saturation. The relative magnetic permeability *µ*_r_ is then obtained from the slope d*M*/d*H* around *H* = 0, which traditionally refers to the magnetic susceptibility *χ* of the material. The relationship between susceptibility and relative permeability is *µ*_r_ = *χ* + 1. The different relative permeabilities for different compression levels are plotted in Figure 7.

The relative permeabilities are slightly larger than 1, meaning that the susceptibilities are slightly larger than 0, denoting a weak magnetic behavior. This is coherent with the low filling factor of the composite of *ϕ*_v_ = 1.35%. It can be observed in Figure 7 that the relative permeability is increasing with the compression *ε*. The change is, however, relatively small; the initial permeability was *µ*_r_ = 1.06 and the largest permeability was *µ*_r_ = 1.11 for deformation of −50%, and the increase in permeability was Δ*µ*_r_ = 0.05. This kind of increase in permeability with compression was also observed by using a permeability meter [15] and similar changes of values were measured. For an isotropic composite with this low filling factor, the Maxwell Garnett approximation [24], is:

(4) $$\mu_{r}\left(\phi_{v}\right)=1+\frac{3\phi_{v}}{1-\phi_{v}}$$

Moreover, the change in permeability Δ*µ*_r_ (*ϕ*_v_, ε) with deformation, assuming a large compressibility of the matrix, can be expressed by [15]:

(5) $$\Delta \mu_{r}\left(\phi_{v},\varepsilon \right)=\frac{-3\phi_{v}}{\left(1-\phi_{v}\right)\left(1-\phi_{v}+\varepsilon \right)}\varepsilon$$

Equation (4) provides *µ*_r_(*ϕ*_v_) = 1.04 and Equation (5), assuming a compression of ε = −0.5, provides Δ*µ*_r_(*ϕ*_v_, ε) = 0.04. These values are relatively close to the values in Figure 7.

This change in permeability with compression is the main sensitive effect in our sensing device, as will be demonstrated in the next section.

### 3.4. Sensing Array

**Figure 8:**
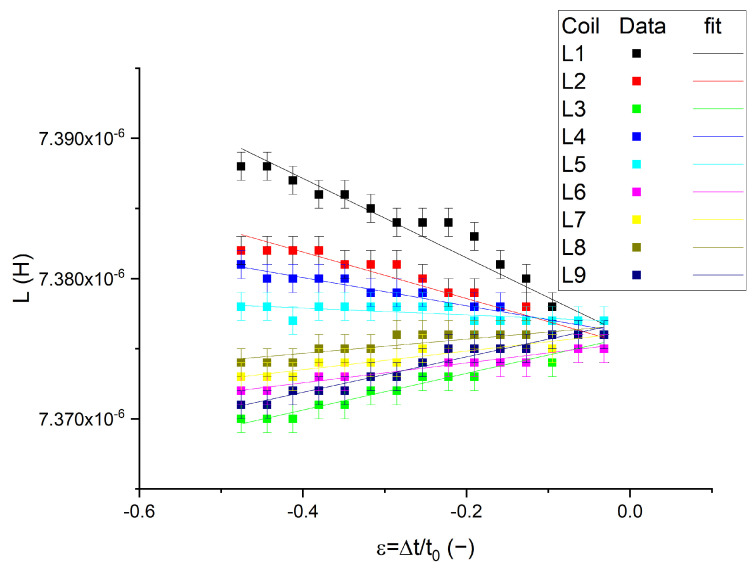
Coil inductance curves versus compression as the composite is deformed above L1.

The first test was the measurement of the inductance of coils as the deformation was applied on the composite above coil L1; see Figure 3b,c. Each coil’s inductance with compression is presented in Figure 8.

Depending on the selected coil for the inductance measurement, the behaviors are different. The initial inductances L_i_(*ε* = 0), where i = 1–9, were slightly different for each coil; this can arise from connections but it is also important to point out that these were all within the measurement uncertainty. More important are the trends of these curves: some have a positive slope, whereas others exhibit a negative slope.

## 4. Discussion

The behavior of the L1 coil inductance with compression provides some indications for the measurement interpretation. This coil is placed just below the compression during the measurements of the curves presented in Figure 8. As the compression became larger, the measured inductance increased: L_1_(ε ≠ 0) > L_1_(ε = 0). This curve behaved as a line in a first approximation, and a linear coefficient *α*_i_ can be defined as:

(6) $$\alpha_{i}=\frac{L_{i}\left(\varepsilon \neq 0\right)-L_{i}\left(\varepsilon =0\right)}{\varepsilon },$$

In the case of the coil inductance L1, the coefficient *α*_1_ was negative. In Figure 7, the compression was inducing an increase in magnetic permeability. The inductance of a coil is proportional to the magnetic flux detected, so it is proportional to the magnetic permeability of the nearby magnetic layer. The composite increased its permeability as it was compressed, so the inductance coil placed below the compressed magnetic layer increased as well. This is coherent with the negative coefficient *α*_1_. The negative value of coefficient *α*_i_ implies a compression.

The other coil inductances that presented a negative slope are L2, L4, and L5, as seen in Figure 8. These coils are located close to coil L1. Thus, it is thought that the composite above these coils was also under compression. To follow this thought, the negative coefficients α_1_, α_2_, α_3_, and α_4_ have amplitudes as |*α*_1_| >|*α*_2_| ~ |*α*_4_| >|*α*_5_|. When looking at the disposition of the coils, coil L1 was placed directly under the applied compression, coils L2 and L4 were equidistant to L1, and coil L5 was located a little farther. As the deformation was applied at point A, it is natural that the deformation was the largest at point A and decreased its amplitude with an increasing distance. This means that the *α*_i_ were actually mapping the local deformation amplitude.

**Figure 9:**
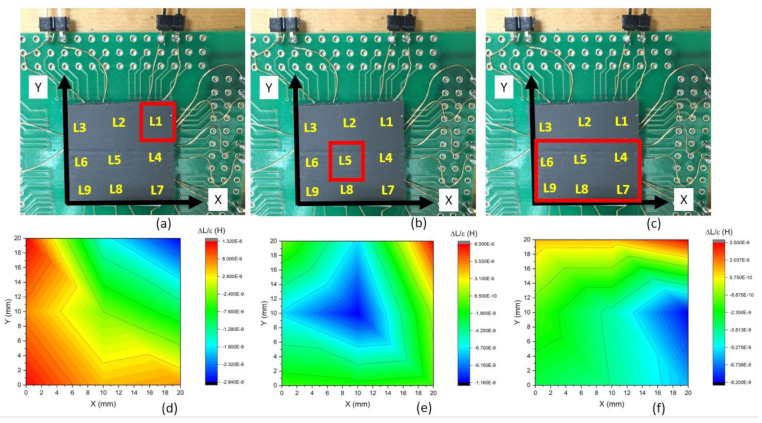
Applied compressive displacement localization (**a**–**c**) and the resulting α(x,y) map (**d**–**f)**: blue and green refer to negative coefficient values (compression), whereas yellow and red refer to positive coefficient values.

By constructing a map of those coefficients according to the position of their respective coils, the position of the compression applied on the composite can be detected. For instance, Figure 9a is the real image of the composite with the area of applied force denoted by a red square. The resulting measurement of the α_i_ coefficients was then interpolated into an *α*(x,y) map, and this *α*(x,y) map is shown in Figure 9d.

The resulting *α*(x,y) map for compression above coil L1 shows two zones: a blue-green area and a yellow-red area. According to the color scale bar, the blue-green color corresponds to *α*(x,y) < 0. The area with low α corresponded well with the position of applied deformation. A second test, where the compression point was located above L5, is presented in Figure 9b, and the measured *α*(x,y) map is shown in Figure 9e. A similar blue-green area was observed at the compression point. Lastly, a wider area was compressed as shown in Figure 9c, with similar results as observed in Figure 9f with a little gradient in the x-direction, suggesting that the sample was not perfectly flat

**Figure 10:**
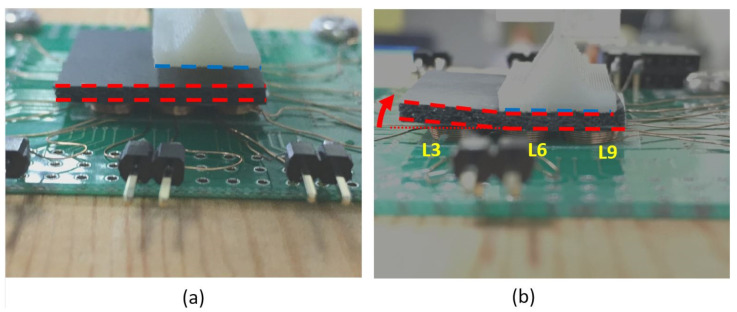
Application of the deformation for the case presented in Figure 7; (**a**) before the application of the deformation and (**b**) after the application of the deformation.

Finally, the coefficients *α*(x,y) > 0 were located far from the compression area, as seen in Figure 9. This was not resulting from an expansion of the material in this area. Instead, it was lifted as observed in Figure 10.

It was optically observed that the composite was mainly compressed where the compression was applied, and it was lifted at the area where it was not compressed (Figure 9b) because the composite was placed on the coil array (Figure 10a) without fixing. The air gap (Figure 10b), created between the composite and the coil, has a magnetic permeability of *µ*_r_ = 1, so the coil detected a lower effective permeability in its neighborhood. This gap then increased as the deformation grew stronger, making the measured coefficient *α*(x,y) > 0 on this area. In this presented experiment, the positive *α* was not necessarily because of the dilatation of the material. This issue can be solved by gluing the magnetic layer onto the coil surface. This was not performed in these experiments because other samples might be tested in future experiments.

**Figure 11:**
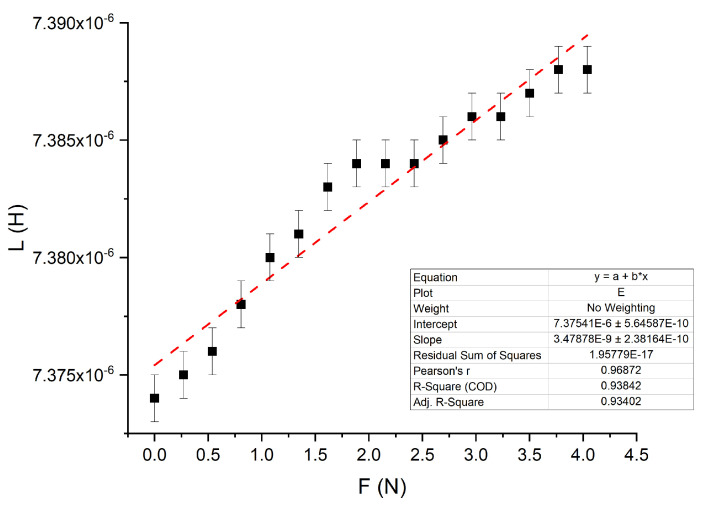
Inductance versus applied force on L1. Broken line is a linear approximation with the parameters shown in the inset.

Finally, the locally applied force can be measured using this system. By combining the stress-strain curve presented in Figure 4, and by scaling it with the coil surface (*A* = πd^2^/4), the local force can be calculated. The inductance behavior of L1 with the deformation, extracted from Figure 8, is then plotted as a function of the local force in Figure 11.

The presented experimental setup could detect local deformation and the corresponding applied forces. By approximating a linear dependency and assuming a measurement error of 1 nH, a force resolution of 0.29 N could be achieved in a measurement range of 0–4.5 N.

## 5. Conclusions

In this article, a magnetic composite was used as an active material sensible to deformation and force. The magnetic composite consisted of a porous (45%) elastomer filled by magnetic particles (1.35%). A sample was shaped in a 3 × 20 × 20 mm^3^ layer. As it experienced uniaxial compression, its magnetic permeabilities were increased, especially the permeability. This change in permeability was successfully detected by a coil array. The stress versus strain curve in compression was recorded, and Young’s modulus was measured. The applied deformation could be converted into applied force. A deformation was locally applied to the magnetic layer. The amplitude of deformation was recorded up to 50% in compression. A map of the inductance change was constructed, giving information on the surface affected by an applied deformation or force.

This system has shown sensibility to (1) the amplitude of the deformation (or force) and (2) to the point of application of this deformation or force. The system was capable of detecting and quantifying a local force applied.

The whole system was compact, as the magnetic layer was 3 mm thick and the coil 2 mm in height, with a diameter of 6 mm. This system is very light and easy to construct. It is believed that it can be convenient for tactile sensing in robotics science.

## Figures and Tables

**Table 1:** Characteristics of transition bands of PU according to references.

Wavenumber (cm^−1^)	Vibration Group	Vibration Type
3440	Free N-H	Stretching ^1,2^
3330	H-bonded N-H	Stretching ^1,2^
2890	CH_2_	Asymmetry stretching ^2^
2850	CH_2_	Symmetry stretching ^2^
1709	Free C=O	Stretching ^1^
1728	H-bonded C=O	Stretching ^1^
1597	Benzene ring	Framework vibration ^2^
1537	N-H	Bending ^2^
1412	CH_2_	Bending ^2,3^
1373	CN	Stretching ^3^
1225	CO	Stretching ^2^

^1^ [20], ^2^ [21], ^3^ [22].

## Data Availability

Not applicable.
